# A UPLC-ESI-MS/MS Method for Simultaneous Quantitation of Chlorogenic Acid, Scutellarin, and Scutellarein in Rat Plasma: Application to a Comparative Pharmacokinetic Study in Sham-Operated and MCAO Rats after Oral Administration of *Erigeron breviscapus* Extract

**DOI:** 10.3390/molecules23071808

**Published:** 2018-07-21

**Authors:** Siying Chen, Mei Li, Yueting Li, Hejia Hu, Ying Li, Yong Huang, Lin Zheng, Yuan Lu, Jie Hu, Yanyu Lan, Aimin Wang, Yongjun Li, Zipeng Gong, Yonglin Wang

**Affiliations:** 1 State Key Laboratory of Functions and Applications of Medicinal Plants, Guizhou Provincial Key Laboratory of Pharmaceutics, Guizhou Medical University, 4 Beijing Road, Guiyang 550014, China; Siying.chen@kcl.ac.uk (S.C.); limei95314@163.com (M.L.); nhwslyt@163.com (Y.L.); huhejia0608@126.com (H.H.); 15761603576@163.com (Y.L.); mailofhy@126.com (Y.H.); mailofzl@126.com (L.Z.); 18798090340@163.com (Y.L.); hujie51619@sina.cn (J.H.); 2 School of Pharmacy, Guizhou Medical University, 4 Beijing Road, Guiyang 550004, China; 3 Guizhou Provincial Engineering Research Center for the Development and Application of Ethnic Medicine and TCM, Guizhou Medical University, 4 Beijing Road, Guiyang 550004, China; Yanyu626@126.com (Y.L.); gywam100@163.com (A.W.); liyongjun026@126.com (Y.L.)

**Keywords:** *Erigeron breviscapus* extract, UPLC-ESI-MS/MS, cerebral ischemia reperfusion injury, scutellarin, scutellarein

## Abstract

*Erigeron breviscapus*, a traditional Chinese medicine, is clinically used for the treatment of occlusive cerebral vascular diseases. We developed a sensitive and reliable ultra-performance liquid chromatography-electrospray-tandem mass spectrometry (UPLC-ESI-MS/MS) method for simultaneous quantitation of chlorogenic acid, scutellarin, and scutellarein, the main active constituents in *Erigeron breviscapus*, and compared the pharmacokinetics of these active ingredients in sham-operated and middle cerebral artery occlusion (MCAO) rats orally administrated with *Erigeron breviscapus* extract. Plasma samples were collected at 15 time points after oral administration of the *Erigeron breviscapus* extract. The levels of chlorogenic acid, scutellarin, and scutellarein in rat plasma at various time points were determined by a UPLC-ESI-MS/MS method, and the drug concentration versus time plots were constructed to estimate pharmacokinetic parameters. The concentration of chlorogenic acid in the plasma reached the maximum plasma drug concentration in about 15 min and was below the limit of detection after 4 h. Scutellarin and scutellarein showed the phenomenon of multiple absorption peaks in sham-operated and MCAO rats, respectively. Compared with the sham-operated rats, the terminal elimination half-life of scutellarein in the MCAO rats was prolonged by more than two times and the area under the curve of each component in the MCAO rats was significantly increased. The results showed chlorogenic acid, scutellarin, and scutellarein in MCAO rats had higher drug exposure than that in sham-operated rats, which provided a reference for the development of innovative drugs, optimal dosing regimens, and clinical rational drug use.

## 1. Introduction

*Erigeron breviscapus*, a traditional Chinese medicine, is mainly distributed in the Guizhou Province and Yunnan Province. It was originally described in the Yunnan Materia Medica, written during the ancient Chinese Ming Dynasty by Zhian Lan, and has been used for the treatment of hemiplegia and rheumatism pain. Moreover, “Quality standard of traditional Chinese medicine and ethnic medicine in Guizhou Province” indicated that *Erigeron breviscapus* possessed various efficacies, including activating collaterals to relieve pain, eliminating wind and dampness, dispelling cold, and relieving the exterior. Therefore, it has been used in treating apoplectic hemiplegia, chest stuffiness, and pains. In addition, *Erigeron breviscapus* is a common medication for ethnic minorities in the Guizhou Province and has been recorded in Chinese Pharmacopoeia (2015) [1].

It is reported that the types of compounds currently isolated and identified from *Erigeron breviscapus* mainly include flavonoids, caffeic acid esters, and aromatic acids [2,3,4,5]. Among them, flavonoids and caffeic ester compounds are characteristic components of *Erigeron breviscapus*, including compounds such as chlorogenic acid, scutellarin, and scutellarein. Studies have shown that *Erigeron breviscapus* possessed various pharmacological effects [6,7,8,9,10,11,12], including scavenging for free radical and antioxidant damage, dilation of blood vessels, improving microcirculation, reducing brain edema, and inhibiting inflammatory reactions. In recent years, emerging research on *Erigeron breviscapus* has become a spotlight as a result of its significant effects on cerebrovascular diseases. Numerous reports have focused on the chemical and pharmacological effects of *Erigeron breviscapus*, also involving limited pharmacokinetic profiling. However, most pharmacokinetic studies on the active ingredients in *Erigeron breviscapus* were mainly investigated under healthy states [13]. It is worth noting that *Erigeron breviscapus* were mainly used to treat hemiplegia. Therefore, pharmacokinetic studies investigating the active ingredients of *Erigeron breviscapus* under pathological states may provide novel insights into how it could be implemented for clinical use.

Thus, we established the first study using an ultra-performance liquid chromatography-electrospray-tandem mass spectrometry (UPLC-ESI-MS/MS) method for the simultaneous determination of three active ingredients of *Erigeron breviscapus,* namely chlorogenic acid, scutellarin, and scutellarein in rat plasma. Furthermore, the pharmacokinetic differences of chlorogenic acid, scutellarin, and scutellatein were investigated between sham-operated and middle cerebral artery occlusion (MACO) rats after oral administration of *Erigeron breviscapus* extract to identify any dose adjustments that may be required for use in the clinic.

## 2. Results

### 2.1. Method Validation

#### 2.1.1. Specificity

**Figure 1:**
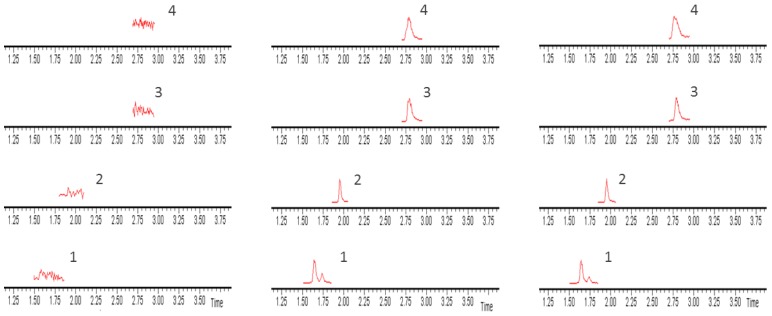
Ultra-performance liquid chromatography-tandem mass spectrometry (UPLC-MS/MS) chromatograms of ingredients in rat plasma: (**a**) blank plasma; (**b**) blank plasma spiked with three components and IS; and (**c**) plasma sample obtained 10 min after intragastric administration of *Erigerin breviscapus* extract (10 g/kg) (1. Chlorogenic acid; 2. puerarin (IS); 3. scutellarin; 4. scutellarein).

The chromatograms of the blank plasma sample, the blank plasma spiked with chlorogenic acid, scutellarin, scutellarein, IS, and plasma samples obtained after oral administration of *Erigeron breviscapus* extract are displayed in Figure 1. The results indicated that the retention times for chlorogenic acid, scutellarin, scutellarein, and IS were 1.64, 2.78, 2.80, and 1.96 min, respectively. No interference from the endogenous substances was observed at the retention time of the analytes and IS.

#### 2.1.2. Calibration Curves and Linearity

The typical equations of calibration curves and linearity ranges for the three analytes are shown in Table 1. The results show that all the correlation coefficients are higher than 0.99, and indicate that the concentrations of the three analytes of chlorogenic acid, scutellarin, and scutellarein in rat plasma correlated well within the linearity ranges.

#### 2.1.3. Accuracy and Precision

The results of the intra- and inter-day precision and accuracy of three analytes in QC samples were shown in Table 2. The RSD (%) values of intra- and inter-day precision for all analytes were not more than 20%, and the RSD (%) values of accuracy of three analytes were within the range of 80.1–117.2%, which demonstrated that the method was accurate, reliable, and repeatable.

#### 2.1.4. Extraction Efficiency and Matrix Effect

As shown in Table 3, the extraction efficiency and matrix effect of the three analytes at three different concentrations and IS were found to be 75.5–102.1%, which indicated the recoveries of the three analytes were consistent, precise, and reproducible at different concentration levels in various plasma biosamples and no significant matrix effect was observed for the three analytes.

#### 2.1.5. Stability

As presented in Table 4, no significant degradation of the three analytes was observed in plasma samples after three freeze-thaw cycles. The three analytes were also stable in a prepared plasma sample solution when placed in the autosampler at 4 °C for up to 24 h. Therefore, the stability could meet the requirements of the analysis method of biological samples.

### 2.2. Pharmacokinetic Analysis

**Figure 2:**
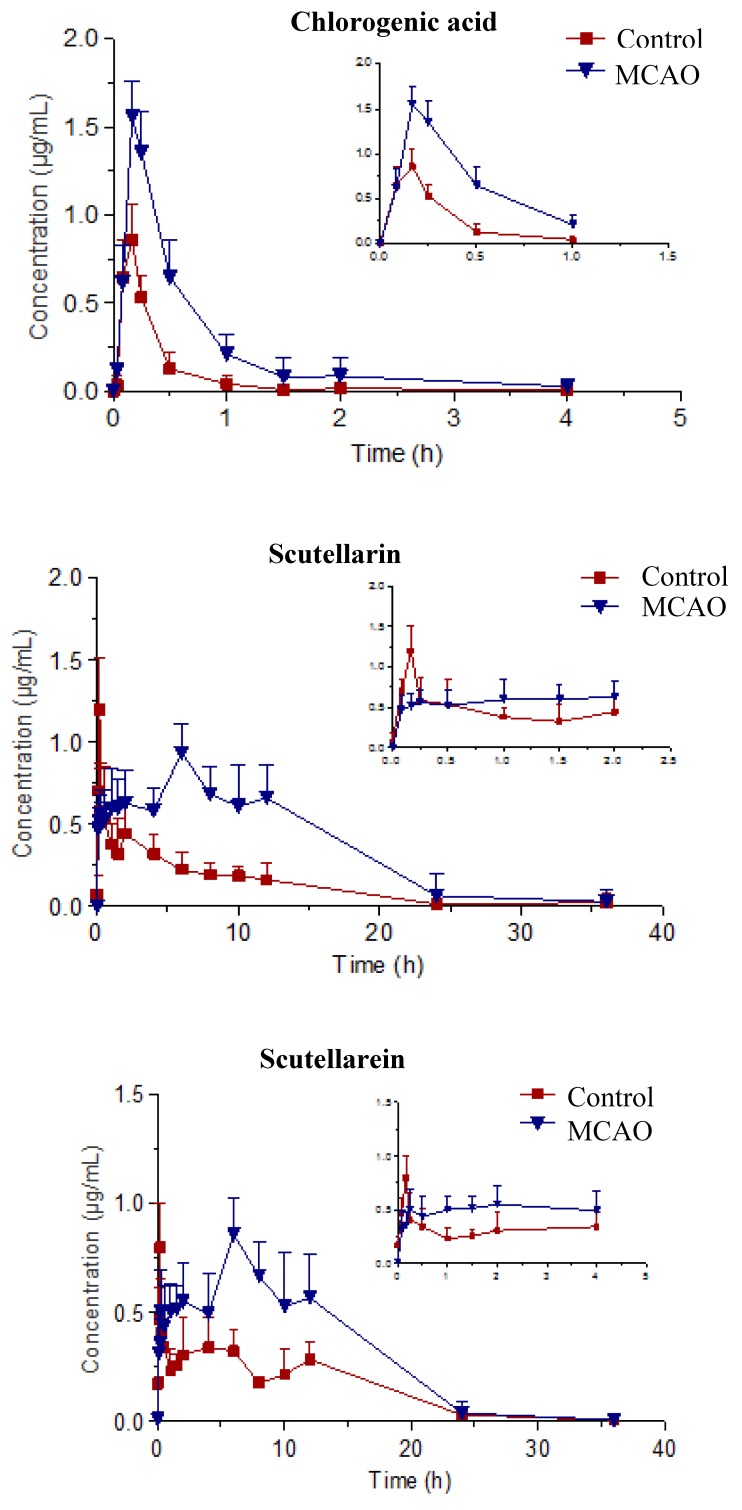
The mean plasma concentration (μg/mL) of chlorogenic acid, scutellarin, and scutellarein vs. time (h) profiles after oral administration of *Erigerin breviscapus* extract (10 g·kg^−1^) in control and MACO model rats. Values were expressed as mean ± *SD* (*n* = 6).

The validated UPLC-ESI-MS/MS method was successfully applied to the pharmacokinetic of three ingredients in rat plasma after oral administration of *Erigeron breviscapus* extract in control and MCAO groups. The mean plasma concentration-time profile is illustrated in Figure 2. Pharmacokinetic parameters were calculated by using DAS 2.0 software (Mathematical Pharmacology Professional Committee of China, Shanghai, China) and a noncompartmental model was used to match the pharmacokinetic process of drug in the rats. Pharmacokinetic parameters of chlorogenic acid, scutellarin, and scutellarein are shown in Table 5, Table 6 and Table 7, respectively.

The concentration of chlorogenic acid in rat plasma reached the maximum plasma concentration in about 15 min and was below the limit of detection after 4 h, when oral administration of *Erigeron breviscapus* extract took place. There were significant differences in pharmacokinetic parameters in control and MCAO rats. The pharmacokinetic parameters of control group were: 0.31 ± 0.14 mg/L·h for ACU_(0–t)_, 0.59 ± 0.19 h for MRT, 48.68 ± 2.77 L/h/kg for CL_Z/F_, 32.07 ± 5.36 L/kg for V_Z/F_, 0.90 ± 0.18 mg/L for C_max_, 0.48 ± 0.15 h for t_1/2_. The pharmacokinetic parameters of the MCAO group were as follows: ACU_(0–t)_, MRT, CL_Z/F_, V_Z/F_, C_max,_ and t_1/2_ of 0.92 ± 0.21 mg/L·h, 0.66 ± 0.23 h, 18.69 ± 2.06 L/h/kg, 11.98 ± 4.45 L/kg, 1.72 ± 0.33 mg/L, 0.63 ± 0.14 h, respectively. The above results demonstrate that chlorogenic acid was able to enter the body quickly, exhibited a relatively rapid absorption and distribution process, and the biological half-life and retention time of the drug in the body were short. Chlorogenic acid changes greatly in vivo between control and MCAO groups; the ACU_(0–t)_ and Cmax of chlorogenic acid in the MCAO group were significantly more than those of the control group. Moreover, the MCAO group had lower clearance and longer half-life, which showed the time of chlorogenic acid in MCAO rats was prolonged and the absorption in vivo was higher than that in control rats.

The vivo process of scutellarin was very complicated. Under the sham-operated and MCAO conditions, there were significant differences in pharmacokinetic parameters. The pharmacokinetic parameters in the control group were 4.63 ± 1.55 mg/L·h for ACU_(0–t)_, 4.23 ± 1.37 h for t_1/2_, 0.14 ± 0.04 h for T_max_, 2.94 ± 1.02 L/kg for CL_Z/F_, and 1.24 ± 0.57 mg/L for C_max_. The pharmacokinetic parameters in MCAO group were 12.93 ± 3.14 mg/L·h for ACU_(0–t)_, 5.75 ± 1.57 h for t_1/2_, 8.67 ± 2.73 h for T_max_, 0.96 ± 0.28 L/kg for CL_Z/F_, and 1.13 ± 0.66 mg/L for C_max_. Scutellarin was rapidly absorbed in sham-operated rats and the maximum plasma concentration was higher than that in MCAO rats. It was absorbed slowly in MCAO rats, and reached the maximum plasma concentration at 8 h. The t_1/2_ and ACU_(0–t)_ of scutellarin changed in control and MCAO rats, the extension of t_1/2_ and significant increase of ACU_(0–t)_ in MCAO rats, which indicated that the absorption of scutellarin in MCAO rats was significantly higher than that in sham-operated rats with longer duration.

There were multiple absorption peaks in the concentration time curve of scutellarein. There were significant differences in pharmacokinetic parameters under the condition of sham-operated and MCAO. The pharmacokinetic parameters of control group were as follows: ACU_(0–t)_, t_1/2_, T_max_, CL_Z/F_, C_max_ of 4.56 ± 1.39 mg/L·h, 4.18 ± 1.01 h, 2.11 ± 4.85 h, 2.6 ± 1.61 L/kg, 0.94 ± 0.47 mg/L, respectively. Correspondingly, in the MCAO group, they were 8.10 ± 2.29 mg/L·h, 11.47 ± 2.83 h, 8.00 ± 2.19 h, 0.88 ± 0.48 L/kg, 1.04 ± 0.67 mg/L, respectively. Compared to the control group, the scutellarein displayed a slow and lasting absorption process and the peak concentration was higher in the MCAO group. Its t_1/2_ in the control and MCAO groups significantly changed, and the extension of t_1/2_ in the MCAO group was more than doubled. Scutellarein was the only component of the three active ingredients of this study that showed a significant difference between physiological and pathological conditions. The ACU_(0–t)_ of scutellarein in the control and MCAO groups showed significant difference as AUC_(0–t)_ was significantly increased in MCAO rats. The results showed that the absorption of scutellarein in MCAO rats was increased, whilst the elimination was slower.

## 3. Discussion

Previous studies have reported that the composition of traditional Chinese medicine was complex, and the material basis for the prevention and treatment of diseases was a comprehensive result of synergistic effects of multiple ingredients [14,15,16]. So far, most studies on the pharmacokinetics of *Erigeron breviscapus* were mainly aimed at the pharmacokinetics of total flavonoids, including scutellarin, but only a few studies exist on the pharmacokinetics of flavonoids and caffeic acid esters in the main active ingredients of *Erigeron breviscapus* [17,18,19,20]. Recent studies have shown that flavonoids and caffeic acid esters in *Erigeron breviscapus* were also active constituents for the treatment of MCAO. Therefore, in this paper, the pharmacokinetics of the representative components of chlorogenic acid, scutellarin, and scutellarein in *Erigeron breviscapus* between sham-operated and pathological rats were studied.

With the emphasis of pharmacokinetics studies of traditional Chinese medicine, a large number of studies showed that the pharmacokinetics characteristics of traditional Chinese medicine were affected by the disease status, which changed the pharmacokinetic process of traditional Chinese medicine in the body by affecting drug-metabolizing enzymes, transport proteins, and endogenous biological factors of the body [21]. In the present study, we found that the pharmacokinetics of chlorogenic acid, scutellarin, and scutellarein in *Erigeron breviscapus* between control and MCAO rats showed significant differences and the bioavailability of three active components of *Erigeron breviscapus* in MCAO rats increased. The reasons for this phenomenon might be explained from two angles.

To begin with, the stress of cerebral infarction enhanced the permeability of the gastrointestinal tract, and eventually increased the gastrointestinal absorption of drugs. The gastrointestinal tract is a vital organ for the digestion, absorption, secretion, and excretion of organisms, and it is also one of the most intense visceral reactions after physiological stimulation. There was a great difference in the gastrointestinal response of individuals to stress, and mild stress can cause abdominal pain, diarrhea, nausea, and vomiting, while severe stress, such as cerebral infarction and head trauma, can lead to stress-related mucosal diseases and gastrointestinal barrier damage [22]. Moreover, brain infarction may stimulate the hypothalamic–pituitary–adrenal (HPA) axis excitability, and, thus, increase the secretion of glucocorticoid and inhibit the secretion of gastric mucus. Meanwhile, autonomic nervous system excitability leads to the decrease of gastric mucus bicarbonate barrier function, the imbalance of apoptosis of mucosal epithelium and proliferation, the disruption of epithelial barriers, and the enhancement of tight junction permeability between mucosal epithelial cells. Moreover, in cerebral infarction, the HPA axis and sympathetic nervous system are activated, and the corticotropin-releasing hormone (CRH) content in the hypothalamus and gastrointestinal tissue are increased, while CRH can inhibit peristalsis of the stomach and small intestine, weakening the motility of the stomach and small intestine, and resulting in the retention of the contents. Studies have found that good general anesthesia and local anesthesia can block the physical impact of tiny trauma and psychological stress response, which precluded the inevitable surgical trauma producing a stress response to the organism after general anesthesia in rats in the process of preparing the MCAO model [22].

Furthermore, cerebral ischemia-reperfusion injury may induce liver injury [23,24]. The stress response after a cerebral ischemia-reperfusion injury can reduce the blood supply of the liver, trigger an inflammatory response, induce hepatocyte apoptosis, and lead to liver damage [25]. It is well documented that the liver is the most significant metabolic regulatory organ in the body, and plays an important role in the biotransformation and elimination of drugs. Hepatic injury induced by cerebral ischemia-reperfusion injury can simultaneously affect the function and activity of liver-metabolizing enzymes, increase or decrease the activity of drug-metabolizing enzymes, and change the metabolism process of drugs, altering the pharmacokinetic process of drugs. For example, Bing [26] argued that the CYP2B in hepatocytes was downregulated after stroke. Yang [27] reported that a sharp decrease of CYP3A in liver cells can be induced by cerebral ischemia in rats, but the antioxidant effect of *Erigeron breviscapus* could alleviate the injury of hepatocytes. It can cause the recovery of CYP3A in hepatocytes and alleviate the injury of hepatocytes after oral administration of *Erigeron breviscapus*.

## 4. Materials and Methods

### 4.1. Materials

Chlorogenic acid (purity >98%), scutellarin (purity >98%), and scutellarein (purity >98%) were purchased from the Beijing Heng Yuan Qitian Technology Research Institute (Beijing, China). Methanol, acetonitrile, and formic acid with HPLC grade were obtained from Merck KGaA Co., Ltd. (Daemstadt, Germany). Distilled water was obtained from Guangzhou Watson Co., Ltd. (Guangzhou, China). All other chemicals and reagents were analytical grade from Beijing Chemical Reagent Co., Ltd. (Beijing, China). Milli-Q Water (Millford, SC, USA) was used throughout the study.

*Erigeron breviscapus* was purchased from Yunnan Medicinal Material Planting Bases of *Erigeron breviscapus*, which were identified by associate Professor Qingde Long, working at the School of Pharmacy of Guizhou Medical University. The *Erigeron breviscapus* extracts were prepared as described previously [28].

### 4.2. Animals

Pharmacokinetic experiments were performed using male Sprague–Dawley rats obtained from Chongqing Tengxin Bio-Technology Co., Ltd. (Chongqing, China) Rats were kept under standard conditions of temperature, humidity, and light. The male rats were housed 6 in a cage with access to food and water ad libitum. All studies were approved by the Animal Ethics Committee at Guizhou Medical University. Animals were randomly divided into two groups consisting of the MCAO and sham-operated groups. The MCAO rat model was induced as described previously [14]. The sham-operated rats experienced the same surgical operations except for no nylon monofilament inserted.

### 4.3. UPLC-MS/MS Instrumentation and Conditions

The UPLC-MS/MS method was performed using a Waters Xevo TQ MS System (Waters, Milford, MA, USA). The system was controlled with Mass LynxV4.1 software (Waters, Milford, MA, USA) for data acquisition and analysis was supplied by Waters Technologies. The LC separation was carried out on an Acquity UPLC BEH C18 column (2.1 mm × 50 mm, id 1.7 mm) and protected by Waters Van Guard BEH C18 column (2.1 mm × 50 mm, 1.7 μm) using a mobile phase consisting of 0.1% formic acid in acetonitrile (A) and 0.1% formic acid water (B). The gradient program was as follows: 0–3 min, 5–25% A and 95–75% B; 3–4 min, 5–90% A and 95–10% B; 4–5 min, 90–5% A and 10–95% B. Efficient and symmetrical peaks were obtained at a flow rate of 0.35 mL/min with a sample injection volume of 1 μL and the column was maintained at 45 °C. The detection of the analytes was used simultaneously with an electrospray negative ionization (ESI–) and electrospray positive ionization (ESI+) and high purity nitrogen served as both nebulizing and drying gas. In the positive ion mode, scutellarin, scutellarein, and puerarin (internal standard, IS) were detected and the optimized parameters were as follows: capillary voltage at 3 kV, cone voltage at 35 V, collision energy 8 eV, and desolvation temperature 350 °C. In the negative ion mode, chlorogenic acid was detected and the optimized parameters were as follows: capillary voltage at 3 kV, cone voltage at 40 V, collision energy 8 eV, and desolvation temperature 350 °C. Nitrogen was used as the desolvation and cone gas with a flow rate of 650 and 50 L/h, respectively. Quantitation was performed using the selected ion recording (SIR) mode of the parent ion, *m*/*z* 353.2 for chlorogenic acid, 463.1 for scutellarin, 286.9 for scutellarein, and 417 for puerarin.

### 4.4. Plasma Samples Preparation

An aliquot of 100 μL plasma sample was spiked with 50 μL 1% formic acid water and 75 μL of puerarin (IS) solution and vortexed briefly. Then, 475 μL of methanol was added to the mixture to be deproteinated, vortex mixed for 1 min, sonicated for 5 min, and centrifuged at 12,000 rpm for 10 min at 4 °C. The supernatant was evaporated by a gentle stream of nitrogen gas. The residue was reconstituted in 200 μL of the mobile phase, followed with centrifugation at 12,000 rpm for 10 min at 4 °C. The supernatant was transferred into an autosampler vial and an aliquot of 1 μL was subsequently injected into the UPLC-MS/MS system for assay.

### 4.5. Preparation of Standard and Quality Control Samples

Stock solutions were separately prepared by dissolving chlorogenic acid (10.08 mg), scutellarin (9.51 mg), and scutellarein (7.88 mg) into methanol to yield a concentration of 1.008 mg/mL chlorogenic acid, 0.951 mg/mL scutellarin, and 0.788 mg/mL scutellarein. A series of working standard solutions were prepared by diluting the stock solution with methanol. All the stock and working solutions were stored at 4 °C and brought to room temperature before use. Quality control (QC) samples were prepared separately in the same process. The QC samples were prepared at 0.025, 0.79, and 3.15 μg/mL for chlorogenic acid; 0.012, 0.37, and 5.94 μg/mL for scutellarin; and 0.019, 0.246, and 3.94 μg/mL for scutellarein.

### 4.6. Method Validation

#### 4.6.1. Specificity

The blank plasma sample chromatogram was conducted under the method of plasma sample preparation that 100 μL of blank plasma taken from rats, except for adding IS. The blank plasma was spiked with chlorogenic acid, scutellarin, and scutellarein and IS chromatogram, and plasma samples obtained after oral administration of the *Erigeron breviscapus* chromatogram were performed in the same fashion.

#### 4.6.2. Calibration Curves and Linearity

The stock solution of chlorogenic acid, scutellarin, and scutellarein was closely weighed and diluted in methanol. Dilutions were prepared to make a series of working solutions. All stocks were stored at –20 °C.

Calibration standards were prepared by spiking the appropriate standard working solutions into 100 μL blank plasma to yield calibration concentrations of 0.0246, 0.197, 0.788, 1.58, and 3.15 μg/mL for chlorogenic acid; 0.0116, 0.0929, 0.371, 1.49, and 5.94 μg/mL for scutellarin; 0.0192, 0.0308, 0.246, 0.980, and 3.94 μg/mL for scutellarein. The calibration curves were constructed by plotting the peak area ratio versus the concentration of the three analytes with linear regression using standard plasma samples at five concentrations. Sensitivity was evaluated by determining limit of detection (LOD) and limit of quantification (LOQ). LOD and LOQ were determined using the signal-to-noise ratio (S/N) of 3:1 and 10:1, respectively.

#### 4.6.3. Accuracy and Precision

The QC samples at three concentration levels of three kinds of constituents of rat plasma were prepared, and operated in parallel according to the above methods of plasma sample preparation; each concentration was analyzed by six replicates, continuous injecting during the day and simultaneous with the standard curve. The precision of intraday and interday was assessed by analyzing six QC samples at each concentration level during the same day and on three consecutive validation days.

#### 4.6.4. Extraction Efficiency and Matrix Effect

The 100 μL of blank plasma was spiked with the QC sample at three concentration levels, each concentration of six replicates, which were prepared according to the above methods of plasma sample preparation and regarded as sample A. Another 100 μL of blank plasma was prepared according to the above methods of plasma sample preparation except for the addition of a mixed standard solution. Sample B was acquired by adding the mixed standard solution and IS into the obtained supernatant and evaporated to dryness, and then the residue was reconstituted with 150 μL of methanol. Sample C was obtained by taking the mixed standard solution and IS to dryness and the residue was reconstituted with 150 μL of methanol. Extraction efficiency was calculated by the peak area ratio (B sample/A sample) and the matrix effect was calculated by the peak area ratio (B sample/C sample).

#### 4.6.5. Stability

The QC samples at three concentration levels of three kinds of constituents of rat plasma were prepared in order to investigate the stability of chlorogenic acid, scutellarin, and scutellarein of processed plasma samples on the autosampler. Plasma samples were processed into the autosampler, and analyzed six samples at each concentration, injected at 6 h respectively. The freeze-thaw stability was tested at three concentration levels by freezing the samples and then thawing them for three times after treatment according to the above methods of plasma samples preparation, then injection was used to detect concentration.

### 4.7. Pharmacokinetic Study

Twelve male Sprague–Dawely rats (280 ± 20 g of body weight) were divided randomly into two groups: the sham-operated and MCAO model with six rats in each group. Jugular vein catheterization was performed on the sham-operated group, and jugular vein intubation and middle cerebral artery ischemia reperfusion injury model was operated on the MCAO model. The *Erigeron breviscapus* extract solution with a dose of 10 g·kg^−1^ was orally administered after surgery at 24 h. The 0.3 mL of blood samples was collected from the jugular vein into centrifuge tubes coated with heparin before administration and at 0.083, 0.167, 0.25, 0.33, 0.5, 1, 2, 4, 6, 8, 10, 12, 24, and 36 h after administration. The plasma was separated by centrifugation at 4500 rpm for 3 min and the 100 μL of supernatant was extracted, then stored at –20 °C until analysis.

### 4.8. Pharmacokinetic Data Processing

The plasma concentration versus time profiles was analyzed using the DAS2.0 data processing software provided by the Mathematical Pharmacology Professional Committee of China. The noncompartmental model was employed to estimate the following pharmacokinetic parameters: terminal elimination half-life (t_1/2_z), area under the plasma concentration vs. time curve from zero to last sampling time (AUC_0–t_) and infinity (AUC_0–∞_), apparent volume of distribution (V_Z/F_), total body clearance (CL_Z/F_), and mean retention time to last sampling time (MRT_0–t_) and infinity(MRT_0–∞_). The maximum plasma concentration (C_max_) and the time of maximum plasma concentration (T_max_) were observed directly from the measured data.

Statistical analysis between two groups was performed by SPSS 18.0 (Chicago, IL, USA) using an independent sample T-test; a *p* value less than 0.05 was considered statistically significant for the test, and all data were presented as means ± standard deviation (SD).

## 5. Conclusions

We have developed a sensitive and reliable UPLC-ESI-MS/MS method for simultaneous quantitation of chlorogenic acid, scutellarin, and scutellarein—the main active constituents in *Erigeron breviscapus*. We were able to compare the pharmacokinetics of these active ingredients in sham-operated and MACO rats orally administrated with *Erigeron breviscapus* extract. We found that the pharmacokinetics of scutellarin, scutellatein, and chlorogenic acid in *Erigeron breviscapus* between sham-operated and MCAO rats that existed were significantly different and the bioavailability of the three active components of *Erigeron breviscapus* in MCAO rats increased. This study will have broad implications and may inform dosing regimens for clinical use.

## Figures and Tables

**Table 1:** The mean values of regression equations of the three compounds.

Analyte	Linear Regression Equation	R^2^	Linear Ranges (μg/mL)	LOQ (μg/mL)	LOD (μg/mL)
Chlorogenic acid	Y = 0.333*X* + 0.0162	0.9988	0.0246–3.15	0.0246	0.0112
Scutellarin	Y = 0.140*X* − 0.0046	0.9992	0.0116–5.94	0.0116	0.0042
Scutellarein	Y = 0.409*X* + 0.0435	0.9995	0.0192–3.94	0.0192	0.0075

**Table 2:** The accuracy, intra-, and inter-day precision of the three analytes in rat plasma ($\bar{x}$ ± *SD*, *n* = 6, 3 days).

Analyte	Concentration of Analyte (μg/mL)	Mean ± SD (μg/mL)	Accuracy (%)	Interday Precision RSD (%)	Intraday Precision RSD (%)
Chlorogenic acid	0.025	0.025 ± 0.004	103.0 ± 16.7	16.2	17.1
	0.79	0.79 ± 0.059	99.9 ± 7.5	7.5	5.3
	3.15	3.14 ± 0.136	99.6 ± 4.3	4.3	6.8
Scutellarin	0.012	0.014 ± 0.003	117.2 ± 21.9	18.6	14.5
	0.37	0.37 ± 0.02	100.1 ± 5.3	5.3	3.9
	5.94	5.82 ± 0.278	98.0 ± 4.7	4.8	9
Scutellarein	0.019	0.013 ± 0.002	80.1 ± 10.2	15	13.3
	0.246	0.243 ± 0.05	98.8 ± 2.0	2.1	7.9
	3.94	3.99 ± 1.179	101.3 ± 3.0	3	5.7

**Table 3:** The mean recoveries and matrix effects of the three analytes in rat plasma ($\bar{x}$ ± *SD*, *n* = 6).

Analyte	Concentration of Analyte (μg/mL)	Extraction Recovery (%)	RSD (%)	Matrix Effect (%)	RSD (%)
Chlorogenic acid	0.025	80.7 ± 10.0	12.4	86.8 ± 2.0	2.3
	0.79	96.7 ± 12.9	13.3	91.4 ± 4.8	5.3
	3.15	80.9 ± 6.1	7.5	91.2 ± 3.5	3.8
Scutellarin	0.012	101.1 ± 4.0	4	93.0 ± 7.3	7.8
	0.37	102.1 ± 16.6	16.3	90.6 ± 9.0	9.9
	5.94	85.8 ± 13.6	15.9	89.3 ± 1.6	1.8
Scutellarein	0.019	80.7 ± 14.8	18.3	98.9 ± 14.3	14.5
	0.246	79.7 ± 5.9	7.4	88.6 ± 4.9	5.5
	3.94	80.3 ± 5.9	7.3	90.9 ± 1.6	1.8

**Table 4:** The stability in autosampler for 6 h, three freeze-thaw cycles of the three compounds in rat plasma ($\bar{x}$ ± *SD*, *n* = 6).

Analyte	Concentration of Analyte (μg/mL)	Sampler 6 h			Three Freeze-Thaw		
		Mean ± SD (μg/mL)	Accuracy (%)	Precision (RSD, %)	Mean ± SD (μg/mL)	Accuracy (%)	Precision (RSD, %)
Chlorogenic acid	0.025	0.025 ± 0.01	101 ± 14.8	14.5	0.021 ± 0.002	84.0 ± 7.7	9.1
	0.79	0.76 ± 0.027	96.9 ± 3.4	3.5	0.73 ± 0.009	93.1 ± 1.1	1.2
	3.15	3.13 ± 0.131	99.5 ± 4.1	4.2	3.04 ± 0.070	96.5 ± 2.2	2.3
Scutellarin	0.012	0.011 ± 0.001	97.1 ± 5.9	6	0.01 ± 0.001	87.9 ± 3.1	3.5
	0.37	0.37 ± 0.020	100 ± 5.3	5.2	0.34 ± 0.016	91.1 ± 4.2	4.6
	5.94	5.86 ± 0.222	98.6 ± 3.7	3.8	5.67 ± 0.217	95.5 ± 3.7	3.8
Scutellarein	0.019	0.018 ± 0.002	94.1 ± 2.1	2.2	0.015 ± 0.003	78.5 ± 9.8	17.6
	0.246	0.246 ± 0.007	100 ± 3.1	3.1	0.24 ± 0.015	96.1 ± 0.6	0.6
	3.94	4.02 ± 0.165	102 ± 4.2	4.1	3.83 ± 0.025	97.3 ± 0.6	0.7

**Table 5:** The pharmacokinetic parameters of chlorogenic acid after intragastric dosing 10 g·kg^−1^ of *Erigerin breviscapus* extract to rats ($\bar{x}$ ± *SD*, *n* = 6).

Pharmacokinetic Parameters	Unit	Chlorogenic Acid	
		Control	MCAO
AUC_(0–t)_	mg/L·h	0.31 ± 0.14	0.92 ± 0.21 *
AUC_(0–∞)_	mg/L·h	0.31 ± 0.14	0.96 ± 0.28 *
MRT_(0–t)_	h	0.59 ± 0.19	0.66 ± 0.23
MRT_(0–∞)_	h	0.60 ± 0.28	0.77 ± 0.28
t_1/2_z	h	0.48 ± 0.15	0.63 ± 0.14
T_max_	h	0.17 ± 0.07	0.19 ± 0.04
CL_Z/F_	L/h/kg	48.68 ± 2.77	18.69 ± 2.06 *
V_Z/F_	L/kg	32.07 ± 5.36	11.98 ± 4.45 *
C_max_	mg/L	0.90 ± 0.18	1.72 ± 0.33 *

* *p* < 0.05 compared with control group.

**Table 6:** The pharmacokinetic parameters of scutellarein after intragastric dosing 10 g·kg^−1^ of *Erigerin breviscapus* extract in the rats ($\bar{x}$ ± *SD*, *n* = 6).

Pharmacokinetic Parameters	Unit	Scutellarein	
		Control	MCAO
AUC_(0–t)_	mg/L·h	4.63 ± 1.55	12.93 ± 3.14 **
AUC_(0–∞)_	mg/L·h	4.69 ± 1.67	13.89 ± 3.48 **
MRT_(0–t)_	h	7.29 ± 2.12	9.77 ± 2.55
MRT_(0–∞)_	h	7.63 ± 2.31	10.57 ± 3.09
t_1/2_z	h	4.23 ± 1.37	5.75 ± 1.57
T_max_	h	0.14 ± 0.04	8.67 ± 2.73 **
CL_Z/F_	L/h/kg	2.94 ± 1.02	0.96 ± 0.28 **
V_Z/F_	L/kg	16.79 ± 4.51	9.66 ± 3.55 *
C_max_	mg/L	1.24 ± 0.57	1.13 ± 0.66

* *p* < 0.05, ** *p* < 0.01 compared with control group.

**Table 7:** The pharmacokinetic parameters of scutellarein after intragastric dosing of 10 g·kg^−1^ of *Erigerin breviscapus* extract to rats ($\bar{x}$ ± *SD*, *n* = 6).

Pharmacokinetic Parameters	Unit	Scutellarein	
		Control	MCAO
AUC_(0–t)_	mg/L*h	4.56 ± 1.39	8.1 ± 2.29 *
AUC_(0–∞)_	mg/L*h	5.58 ± 1.81	8.49 ± 3.17 *
MRT_(0–t)_	h	6.7 ± 1.86	8.51 ± 2.48
MRT_(0–∞)_	h	7.99 ± 2.78	9.49 ± 3.66
t_1/2_z	h	4.18 ± 1.01	11.47 ± 2.83 *
T_max_	h	2.11 ± 4.85	8 ± 2.19 *
CL_Z/F_	L/h/kg	2.6 ± 1.61	0.88 ± 0.48 *
V_Z/F_	L/kg	16.09 ± 9.47	9.85 ± 5.56
C_max_	mg/L	0.94 ± 0.47	1.04 ± 0.67

* *p* < 0.05 compared with control group.
